# Interventions addressing student bullying in the clinical workplace: a narrative review

**DOI:** 10.1186/s12909-019-1578-y

**Published:** 2019-06-21

**Authors:** Althea Gamble Blakey, Kelby Smith-Han, Lynley Anderson, Emma Collins, Elizabeth Berryman, Tim J. Wilkinson

**Affiliations:** 1 Otago Medical School, University of Otago, Dunedin, NZ New Zealand; 2 Bioethics Centre, University of Otago, Dunedin, NZ New Zealand; 3 Otago Polytechnic & Staff Nurse, Southern District Health Board, Dunedin, NZ New Zealand; 4 North Shore Hospital, Waitemata District Health Board, Auckland, NZ New Zealand

**Keywords:** Bullying, Clinical environment, Intervention, Medical student, Nursing student

## Abstract

**Background:**

Student bullying in the clinical environment continues to have a substantial impact, despite numerous attempts to rectify the situation. However, there are significant gaps in the literature about interventions to help students, particularly a lack of specific guidance around which to formulate an intervention program likely to be effective. With this narrative review about student bullying interventions in the clinical learning environment, we examine and draw together the available, but patchy, information about ‘what works’ to inform better practice and further research.

**Methods:**

We initially followed a PICO approach to obtain and analyse data from 38 articles from seven databases. We then used a general inductive approach to form themes about effective student bullying intervention practice, and potential unintended consequences of some of these, which we further developed into six final themes.

**Results:**

The diverse literature presents difficulties in comparison of intervention efficacy and substantive guidance is sparse and inconsistently reported. The final analytical approach we employed was challenging but useful because it enabled us to reveal the more effective elements of bullying interventions, as well as information about what to avoid: an interventionist and institution need to, together, 1. understand bullying catalysts, 2. address staff needs, 3. have, but not rely on policy or reporting process about behaviour, 4. avoid targeting specific staff groups, but aim for saturation, 5. frame the intervention to encourage good behaviour, not target poor behaviour, and 6. possess specific knowledge and specialised teaching and facilitation skills. We present the themed evidence pragmatically to help practitioners and institutions design an effective program and avoid instigating practices which have now been found to be ineffective or deleterious.

**Conclusions:**

Despite challenges with the complexity of the literature and in determining a useful approach for analysis and reporting, results are important and ideas about practice useful. These inform a way forward for further, more effective student bullying intervention and research: an active learning approach addressing staff needs, which is non-targeted and positively and skilfully administered. (331w).

**Electronic supplementary material:**

The online version of this article (10.1186/s12909-019-1578-y) contains supplementary material, which is available to authorized users.

## Background

A substantial proportion of healthcare students worldwide experience bullying in clinical practice [1–4]. Its prevalence, nature and consequences are well documented: one survey indicated 59% of medical students can expect to be bullied by staff (doctors, nurses, allied health, including management) with whom they work, at some time during their clinical training [2].

Evidence suggests any student can suffer bullying in the clinical workplace, and at the hands of any staff member [5–7]. However, senior staff are reported to be the most likely perpetrators, and students of minority ethnicity, gender or sexuality are likely to fare worse [2]. Verbal harassment, gender and racial discrimination, and academic harassment (e.g. withholding a grade in return for favours) are among the commonest recorded bullying acts [8, 9].

### Clarification of terms

We use *student* to represent a learner in the clinical workplace undertaking supervised clinical work in the pursuit of a foundational clinical qualification. We exclude postgraduate learners, as they are often ‘positioned’ differently within a workplace structure.

The potentially complex nature of student *bullying* in the clinical workplace results in it having various definitions, none of which seem widely accepted. Hence, we use a definition which serves the current review’s purpose, and has a specific focus on the undergraduate learner:

Mistreatment, either intentional or unintentional occurs when behaviour shows disrespect for the dignity of others and unreasonably interferes with the learning process. Examples of mistreatment include sexual harassment; discrimination or harassment based on race, religion, ethnicity, gender, or sexual orientation; humiliation; psychological or physical punishment; and the use of grading and other forms of assessment in a punitive manner ([10], p. 706).

A significant body of experience and research explicates the considerable overall harm that healthcare student bullying can cause. The potential severity of this harm, together with its prevalence [2], strongly suggests bullying is an important problem to consider for all concerned. In summary, bullying can harm a victim’s learning and the learning of others in the workplace, influence career choices [11–13], create short and long term mental health issues and lead to self-harm and suicide [14–17]. Student bullying can also be witnessed by, and be distressing to others, the consequences of which might also then impact on the functionality of a clinical service. Together with the bullying of staff more generally, student bullying is a potentially significant threat to quality (e.g. patient outcomes, clinical error), efficiency, levels of job satisfaction, staff retention and turnover [10, 16].

A particular feature of bullying behaviour is that it can become pervasive [18] and dominate workplace culture, and subsequently be ‘passed down’ to further generations of staff and students [9]. Apart from the known impact of role modelling, suggested explanations for this phenomenon are that a bullied person develops a sense of defeatism and ‘learns’ such behaviours themselves [19, 20], and they then fail to develop effective teaching or communication skills, or insight into how their behaviour affects others [21]. Related to this feature is that bullying can be catalysed by workplace conditions [22] particularly at times of resource constraints, major change or other workplace uncertainty [9]. Students are also at a natural ‘disadvantage’ in the clinical workplace, due to potential power differences and misuse of hierarchy, and some perhaps only beginning to develop their capacity for resilience. Thus, the healthcare student can be vulnerable to bullying *and* less well equipped than some others to cope with it [23, 24].

While several reviews of the healthcare literature specifically acknowledge the potential pervasiveness and tenacity of the general bullying problem, many lack detail relative to *student* bullying and how to address it. This is despite bullying being described as a specific and ongoing concern in recent literature and media [25, 26]. For example, Stagg & Sheridan [27] comment on the effectiveness of interventions administered to nurses, with a focus on identifying best practice, but their report includes reviews of interventions undertaken in non-clinical workplaces such as schools, an appreciably different context to the clinical environment. Interventions reviewed therein also lack reference to the specific context of the *adult student learner*. Similarly, a substantial, commissioned review of the National Health Service (NHS, UK) includes a diverse range of non-medical/nursing clinical workplaces such as dentistry [28] and again lacks a significant focus on the student.

Other reviews provide a commentary on nursing bullying research [29] and contain limited descriptions of non-specific recommendations for future practice, e.g. the provision of counselling for victims. D’Ambra & Andrews [30] report specifically about the effect of bullying on the new graduate nurse, and Gallo [31] reports the nature of behaviours in nurse education generally. Some aspects of bullying in the clinical workplace, and some professional groups, receive considerable attention in these reviews. However, there is still little information to guide those currently planning to instigate a programme specifically for staff to improve behaviours around *students in the clinical workplace*. This gap is acknowledged by others, as are inconsistencies in how any interventions are evaluated and reported [27, 32]. While we do find some useful information in the literature, such as advice to administer an intervention before bullying behaviours escalate [33, 34], there is still little detailed guidance to formulate a specific approach.

Emergent evidence from the literature also raises the issue of unintended consequences of a bullying intervention, which suggests the need for a new direction in research. This evidence links some bullying intervention and complaint processes to deleterious ‘adverse effects.’ For example, it has been noted that an intervention can cause staff to disengage from learning, and bullying behaviour can become exacerbated if complaints are handled in certain ways [9, 34]. That is, some interventions and complaints processes might actually be harmful, or create further problems for the student.

Bullying intervention research is thus in need of review regarding the latest thinking and a clearer overall understanding of what might, and might not, be helpful for the healthcare student. We offer a narrative review of this literature with a pragmatic focus to inform those wishing to effectively address student bullying in the clinical environment. This review builds on the work of Fnais [2] which focuses on the persistence and prevalence of bullying in the clinical environment. Now, with the current review we ask:

*What are the features of effective or ineffective interventions aimed at preventing or reducing the student bullying in the clinical environment?*

## Method

We used an approach to narrative review as outlined by Green [35]. This approach includes searching for journal articles and also referencing other sources that researchers view as important to the topic in review. Therefore, we included some authoritative books that included information relevant to our review topic [35]. As many databases exclude such texts, we included Google Scholar in our database search for this purpose.

Our inclusion criteria for academic papers were peer-reviewed English language papers from 1991 to 2017 that described research into interventions undertaken in clinical workplaces to address student bullying, and administered to populations of nursing, medicine and allied health professionals. These dates purposefully include early published/evaluated interventions from a time when ideas about bullying in the clinical workplace began to be developed in earnest. Clinical staff, and the healthcare environment is our specific area of interest because of a lack of in-depth reviews in the current literature about this specialised, but important context.

Exclusion criteria were non-English language articles and those published before 1991, and those not describing any kind of evidence from research. We excluded articles about student-to-student bullying and interventions undertaken in the healthcare student classroom setting. We excluded interventions undertaken in primary healthcare settings (dentistry, optometry, podiatry and general practice) as these working contexts have a very different workplace structure to the hospital, for example, healthcare staff can work in considerable isolation from each other rather than being part of a more clear ‘team’ structure.

In summary, we limited our search to students in the hospital setting because the environment can significantly influence bullying manifestation and type [9]; the student learning environment is unique, as is the positioning of the student within the staff structure in it.

### Literature search in detail

Two researchers searched the literature using specific keywords (see Table 1) developed from our research question. In this selection, researchers were supported by the wider research group members and a research librarian specialist. Keywords were initially developed using a PICO structure [36] (Population, Intervention, Comparison, Outcome), which offers a useful, structured, tailored approach to developing and recording search terms. We modified the PICO formula by adding an extra category of ‘I’ (intervention) so we could ease the administration of searching for descriptions of bullying acts (e.g. to include other key/common words such as mistreatment) and substituted ‘environment’ for ‘comparison.’ Our electronic search crossed seven databases: EMBASE, ERIC, Google Scholar, Medline, Science Direct, Scopus, Web of Science.

**Table 1:** A table which explicates the search terms used for the literature review

P	1		Medical student OR student nurse OR student physiotherapist, student midwife
I	2	AND	OR intervention OR strategy OR education OR staff development OR policy OR professional development OR behaviour modification
Ia	2a	OR	Verbal OR belittlement OR bully* OR sexual harassment OR abuse OR gender OR emotional OR mistreatment OR pimping OR incivility
C	3	AND	Clinical OR, education OR environment OR hospital OR ward
O	4	AND	Prevent OR stop OR reduce OR alleviate OR address OR successful OR unsuccessful OR outcome
		NOT	dental, pharmacy, optometry, podiatry, general practice, generalist, classroom, prison, primary, secondary, high school, parent, youth, girls, boys

Search terms using the PICO method. *P* Population, *I* Intervention (variable of interest) as action (2) or actual intervention (2a) *C* Comparison (we used Environment as it aligns with our topic more accurately t), *O* Outcome

### Stage 1 search term definition (PICO method)

As we progressed with our literature search, using the PICO method (see Table 1 outlining the terms we used), words which were unfamiliar and outside our initial search terms became apparent. For example, e.g., ‘pimping,’ which means deliberately asking a student difficult questions intended to embarrass [12]. Where these words were considered important, they were added to our search criteria and searches re-run.

### Stage 2 terms excluded

We avoided gathering articles outside our remit by adding a ‘NOT’ term search to the original database searches, specifically, we excluded the following under the (P, population) category: dent,* pharmacy, optometry, podiatry, general practice, generalist, parent, youth, girls, boys, classroom, prison, primary, secondary, high school, parent/ing.

### Stage 3 further eliminating articles from the literature search

Independent comparison of Excel spreadsheets allowed the two researchers to remove duplicate and ineligible articles. Specifically, by checking each abstract for general relevance and removing those containing relevant keywords, but ultimately did not match our criteria (e.g. about prevalence only).

### Stage 4 extraction of detail

The researchers went on to extract detailed data from each article, using comparisons of data recorded under the PICO headings, and then by asking the question: what was going on in this clinical workplace? What was done, and how, what were the outcomes, and if measured, how?

### Data extraction and analysis

Because of the diversity of articles identified in the search, the PICO formula for extracting detailed data eventually became less useful, and researchers moved onto using a general inductive thematic approach, as developed by Thomas [37], which allowed us to record additional notes and categories. As analysis of the reviewed papers progressed, themes/data categorisation were identified, discussed, changed, and reviewed by all authors, in an ongoing cycle until consensus and data saturation were reached. Ultimately, after several consultations with all authors, categories developed into the six themes and associated sub-themes.

## Results

**Fig. 1:**
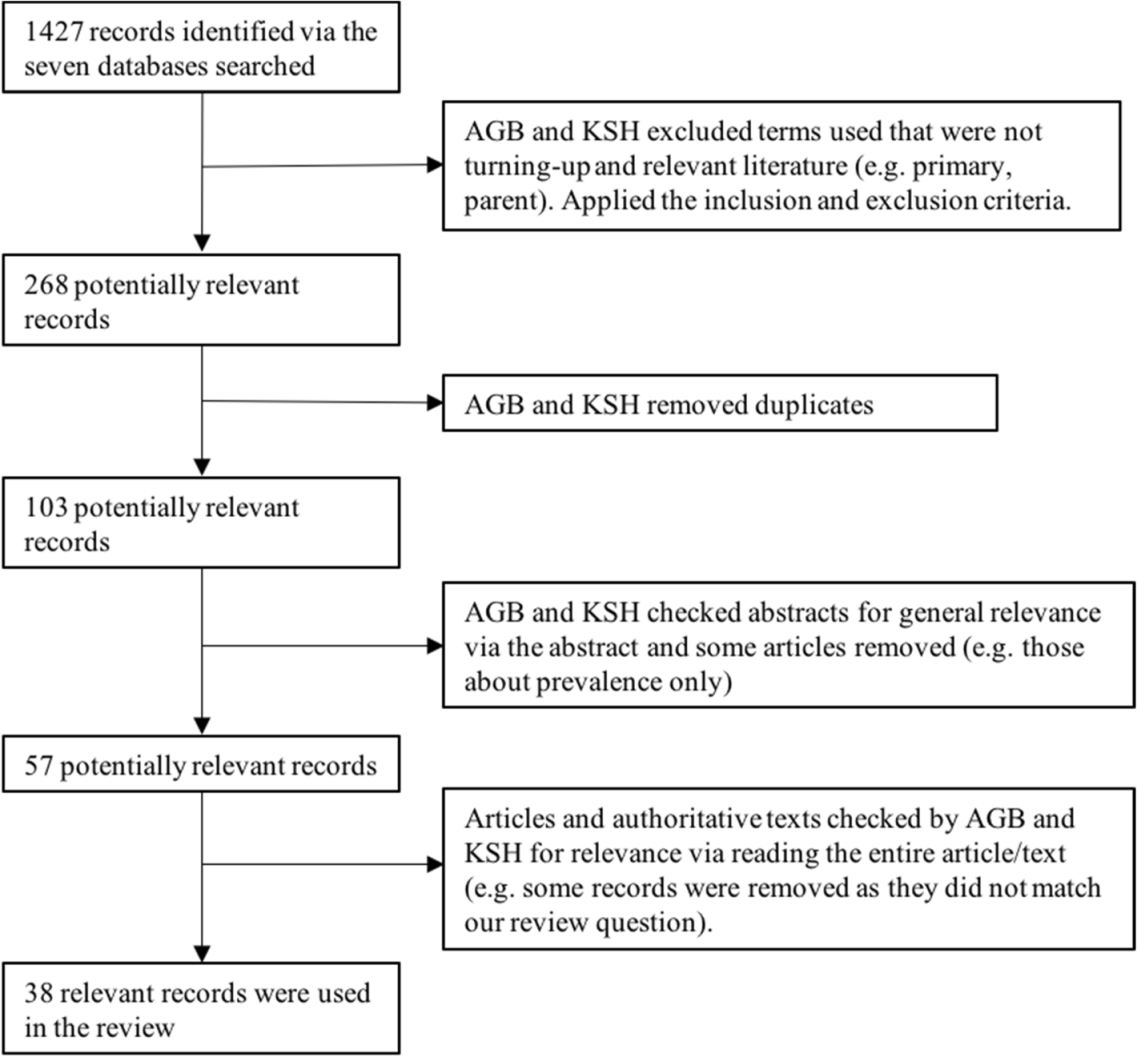
Flowchart of the literature search

A total of 1427 articles were identified, collected and reviewed, from which 38 met our inclusion criteria (Fig. 1); 36 were journal articles and two were authoritative books based on collated research. These two books represent a substantial amount of peer reviewed work, some translated into English. Articles and texts from this search describe quantitative or qualitative data and refer singularly to student bullying, or to both staff and student bullying in the clinical environment.

Despite being peer reviewed, and most containing substantive data, we found some papers were couched more as comments/opinion pieces or described ideas for intervention but not research around these. The following themes and associated sub-themes are derived from all remaining articles and books (Table 2).

**Table 2:** Things to consider for optimal effectiveness when developing and administering a student bullying intervention

1. Understand bullying catalysts	
a) Understand what might cause or catalyse student bullying in a workplace, e.g. resource constraints	
b) Be aware of potential personal issues for staff for whom the intervention is intended e.g. lack of specific training in teaching skills	
c) Tailor the intervention to take these into account (at least)	
2. Staff need: an interventionist to understand what they do, a relationship with the interventionist, and their adult learning requirements addressed.	
a) Understand what staff do and ensure the intervention is easily accessible to them	
b) Ensure the intervention method allows a functional and supportive relationship between staff and interventionist to be established	
c) Teach staff in ways that adults are more likely to learn (active learning) especially if values issues need to be addressed	
3. Policy: necessary but not sufficient	
a) Ensure policy about behaviour is up to date and clearly explicates the complaints process	
b) Ensure policy remit includes staff on adjunct contracts	
c) Ensure staff know about policy and understand how to use it	
d) Ensure management are skilled in managing policy/complaint processes and offer support to *each* employee involved in a complaint	
e) Ensure that potential student/staff bullying is addressed in another way, asides from implementing policy or process, ideally proactively	
4. No targeting specific groups, and aim for saturation	
a) Include diverse staff groups in the intervention and frame it in ways to avoid targeting staff groups/specific people/specific behaviour	
b) Include as many staff as possible (may require several interventions)	
5. Frame the intervention to improve behaviour, not eradicate bad behaviour	
a) Ensure the intervention has a positive, relevant topic/content focused on upskilling	
b) Ensure management overtly support the intervention and participate	
c) Ensure staff are offered long term support in enacting new skills and changing workplace culture	
6. Interventionist teaching and facilitation skills matter	
a) Ensure the interventionist is skilled in teaching with active learning processes	
b) Ensure the interventionist is knowledgeable of how the clinical workplace functions	
c) Ensure the interventionist is aware of the need to keep information confidential	

The articles reviewed are summarised in Additional file 1: Appendix A illustrating how evidence from the articles and books contributes to our resultant six-theme framework. In summary, our final research question was:

*What are the features of effective or ineffective interventions aimed at preventing or reducing student bullying in the clinical environment?*

Themes were:

- Understand bullying catalysts
- Establish a relationship between the staff and the interventionist so that staff needs are understood
- Policy, necessary but not sufficient
- Aim for saturation rather than targeting specific groups
- Frame the intervention to improve behaviour, not eradicate bad behaviour
- Interventionist teaching and facilitation skill matters

We now describe, and then discuss each theme.

### Understand bullying catalysts

Under this theme we describe findings suggesting an intervention should be designed only after developing an understanding of potential catalysts for bullying in a workplace. This suggestion arises from noting bullying can be a consequence of a poor quality work environment and/or personal factors such as values [20, 34, 38, 39]. Because some workplace and personal factors make student bullying more likely, identifying and understanding these is important before deciding what an intervention should exactly contain (e.g. upskilling in communicating well at busy times).

Relevant papers informing this theme were a retrospective analysis of departmental incident reports [22], a descriptive piece based loosely on several pieces of research [39] and a model for the interpretation of potentially complex workplace behaviours and catalysts, developed from a review of nursing literature [38]. Overall, most articles stress the need for an early workplace intervention which takes into account what might cause bullying, rather than one which aims to simply ‘cure’ an existing problem. Examples of bullying catalysts in the clinical environment include:

- environmental - monotonous or heavy workload, job insecurity, long hours, specialism, high-technology, high responsibility [40] and lack of job control [41];
- personal - lack of social support [41] or training, say, in teaching or skills of clinical practice [22, 41] and being stressed or burnt-out [16, 22].

In the light of the variation and significance of possible bullying catalysts, an across-the-board intervention (‘silver bullet’) is unlikely to effectively address student bullying in all workplaces. Some further suggest that failure to identify and address catalysts could itself be understood as a way to ‘facilitate’ or perpetuate bullying [40, 41].

### Establish a relationship between the staff and the interventionist so that staff needs are understood

We use three sub-themes (a, b, c) to describe findings regarding staff who take part in a bullying intervention. In contrast to bullying *causes* or *catalysts* described above, texts reviewed here also introduce references to possible deleterious *effects* of a bullying intervention itself.

#### Staff need an interventionist to understand what they do

Clinical work has a unique context, to include specific tasks and accompanying duties, responsibilities and stressors. As we describe above, an understanding of this context has been shown to be important to understanding whether these factors might cause, catalyse or help bullying to persist in a specific workplace.

However, it has also been found that an interventionist needs to develop a knowledge and understanding of what exactly staff do and a department’s clinical function within the health system, again, in order to offer appropriate, applicable content, but also to ensure the offering is accessible [33, 39, 42–44]. An example of such an understanding might be an intervention should be tailored in a way that staff can learn skills specifically for use in an operating theatre, and for it to be offered at times/places accessible to staff ‘tied’ to such a workplace. An interventionist *taking time* to acquire such knowledge, and to develop an intervention around it, can help staff to engage in learning as it indicates a degree of respect for those taking part and understanding of their personal situation [34].

#### Staff need a relationship with the interventionist

Administering a bullying intervention is not a straightforward matter of designing and administering a programme with particular content. It has also been found staff need opportunity to develop a functional relationship with the interventionist, in order to achieve learning outcomes. Such a relationship is needed for the interventionist to signify respect for staff and to help staff view an interventionist as credible - both ultimately enhancing engagement in learning. A specific suggestion for developing such a relationship is that an interventionist positions themselves as someone offering help, rather than one delivering information or imposing a view; some authors stress overall staff engagement can depend on the ‘approval’ or credibility of an interventionist, obtained by such a relationship [39–41, 45].

Slightly aside to the above, two texts also discuss ‘relationship’ in terms of a broad requirement to maintain a functional relationship with both victim and bully, as would need to be the case where (for example) full, and truthful information about a bullying incident is being sought by an interventionist. Authors argue that relationship can be key in such cases, as ultimately any effective intervention would need to be based on obtaining such information [1, 34].

#### Staff need their adult learning needs to be addressed

Clinical staff are adult learners, and as such, have specific learning needs, and for a variety of reasons. While couched mostly positively, this sub-theme contains several references which also strongly infer what might happen if an inappropriate teaching method is used.

One important finding in this sub- theme is that adult learners are unlikely to respond well to ‘being lectured’ or ‘told’ as would likely be their experience in a ‘lecture’ about behaviour. As in adult education more generally, active learning methods have become widely accepted to cater specifically to these learners and generally enhance engagement and learning [46–48]. For example, replacing a lecture about behaviour with active learning as a small group discussion can help staff engage in dialogue specific to their own context, events and experiences, and can help staff to reflect on their practice in a ‘safer’ environment - arguably an essential factor in positive behaviour change. Active learning in small group discussion has also been shown to help staff generally explore their own behaviours, and in relation to effects of bullying [49–52] and, especially for older staff, to feel recognised for their existing skills and knowledge [20].

Thus, the achievement of a bullying intervention’s learning outcomes can depend on how it is administered, what ‘is in’ the programme (and described in other sections) and at times, on emotional factors such as a staff member feeling respected and valued. Schoonbeek & Henderson report a participant’s positive, transformational experiences of active learning methods used as part of a bullying intervention:

*…*the tools provided to us…have the power to change an age old culture embedded in blame and inequality. I have had to examine my own practice and ensure that I adopt an attitude that reflects the behaviours that I expect from my colleagues ([52], p. 47).

Further claims about the importance of active learning processes centre around values, attitudes or behaviours, all of which are potentially complex, challenging and delicate to discuss and change. Because of this, some staff might benefit from the support offered by being in close contact with a small group of colleagues, a common pre-requisite for active learning [34, 45]. While one author notes hardened or ‘recidivist’ bullies might still be untouched by many such kinds of intervention [53], others argue that closeness and support, as offered by most active learning strategies, should indeed be obligatory. This opinion is based on the supposition that developing staffs’ reflective thinking and values are central goals for most interventions [34]. Here, Lucey & Souba summarise how values change is crucial for most staff to change their behaviour:

…enforcing rules and throwing resources at an adaptive challenge won’t solve the underlying problem, although those steps might temporarily mitigate the symptoms. The solution generally requires changes in the individual ([45], p. 1019).

Some authors also indicate ongoing support might be required to learn to enact the skills learned from an intervention, as these might take time to develop, apply and practise, especially where a negative workplace culture is engrained [33, 54]. While a specific timeframe is not offered, some recommend further exposure to a program of intervention possibly six months after the initial intervention [54]. The utility of following-up active learning is supported by others who indicate that widespread change is unlikely in the short term, but would become more so with support over time [33].

In summary, active learning methods do not *guarantee* behaviour change: participant staff might learn well but fail to action their new learning if they are the only one doing so [55]. This idea seems to reinforce the notion raised in sections (a) and (b) about the support active learning offers for staff group members to enact new skills or values in the working environment. There may be no ‘silver bullet’ to cure bullying, but tailored, multifaceted, repeated or ongoing interventions are likely to do better.

### Policy: necessary, but not sufficient

Policy about behaviour is an important feature of a clinical workplace, but on its own has been shown to be generally ineffective for changing it; policy is of course necessary, to set out standards and limits of staff behaviour [1, 22, 40, 41, 56–59], legitimise a complaint [9] and explicate complaints processes, and overall to promulgate professional values [18, 57]. While in some cases [59] policy has been shown to be more effective where used in conjunction with a structured and easy to follow action plan for management, authors universally stress that policy alone, or instigated as a bullying ‘intervention’ is unlikely to affect staff behaviour [55, 57, 58, 60–66]. Authors justify failure variously: lack of engagement with policy (e.g. ‘it’s not about me’), failure to know it exists or to understand how to use it [9], or because bullying can have complex causes and different manifestations [58]. Johnson [58] illustrates such potential with the use of an ‘ecological model’ of potential bullying causes. Despite these complexities, developing a new or revised policy is one of the more common workplace responses to a bullying complaint.

Policy about behaviour and complaints processes has been shown to be generally ineffective because bullying is notoriously under-reported [66]. This means policy is not enacted as often as the bullying acts are committed. Kohut [63] estimates that 40% of bullying victims fail to verbally inform their employer, let alone formally complain. Failure to report bullying can result from a lack of understanding that bullying is unacceptable, or differing views of what constitutes bullying [9] or dismissing behaviour as idiosyncratic [67], fear of career/academic ‘suicide’ [68] or because of a perceived lack of confidentiality during the complaints process (especially for sensitive issues, e.g. sexual harassment) or, sadly, an understanding that processes will be, or have been, administered unfairly or improperly [68].

Policy can also be rendered ineffective in cases where management fail to take action, such as rejecting responsibility to address bullying complaints, what some call ignoring the ‘elephant in the room,’ ([69], p. 1492). This phenomenon has been shown to be especially pronounced where an accused is on a dual academic/clinical contract (and a complaint ‘dodged’), or where they possess desirable skills or inhabit a leadership role [9, 68–70]. In summary, policy about staff behaviour can be ineffective as an intervention on its own, or at times when it is not accessed, understood, followed, or effectively actioned.

### Deleterious effects of policy/process

Emerging evidence reveals some behaviour policy/complaints processes, particularly requests to keep a complaint confidential, can have deleterious effects on staff and resultant behaviour [34]. Such a request can mean the accused feels, or is seen by others as ‘guilty’ prior to investigation, and precludes that person accessing collegial support. Requests for confidentiality usually aim to avoid skewing investigation, say, by ensuring an accused staff member doesn’t inappropriately ‘gather supporters’ [9, 34]. However, it has been found such a request can be experienced as ‘marginalisation,’ a practice similar to that employed by some bullies. Importantly, these experiences have been reported to exacerbate behaviours because the accused may interpret the request as management’s implicit approval of bullying tactics [34]. Such a perception can be heightened where management seem to lack the requisite skill in administering disciplinary processes [9, 23, 34].

Instigation of policy processes might infer someone is ‘guilty,’ even prior to formal investigation may also be detrimental to outcomes, because it prevents a bully from seeking help in the first instance. It immediately makes the process punitive rather than supportive. For example, staff who recognize their behaviour as problematic might fail to seek help because they understand that a ‘guilty’ label would be then bestowed, and negatively affect their reputation [34]. McGregor [34] stresses properly enacted bullying policy should actually entail skilfully nuanced practices that avoid such inference, but instead withhold judgment and compassionately and respectfully offer support to both victim and accused. This author emphasises such practice is especially important, given the understanding an employer has a duty of care for the wellbeing of *each* employee and accusations can be unfounded, exaggerated or explanations incomplete [34].

### Aim for saturation rather than targeting specific groups

Having explained how an intervention might respond to a workplace’s context to assist in engaging staff in learning, we find indications that a relatively ‘broad’ approach, including all staff, can also optimise engagement [52] particularly where positive relationships begin to be reinforced or forged between professional groups [71]. For example, an approach which crosses disciplines and groups of people, and aims to include everyone in developing a new work culture around students – as opposed to one aimed singularly at ‘troublemakers’ or ‘the nurses.’

As part of a non-targeted approach, it has also been shown it can be helpful for management staff to ‘be seen to’ actively participate in any workplace intervention, as well as to support it via provision of resources and by releasing staff to attend. Management participation is also important because these staff are not immune to bullying behaviours, and in some cases are central protagonists. Either way, ‘what management do’ (role modelling and showing they are learning and supporting other staff) has been shown to vastly influence workplace culture and any intervention that aims to change it: a student bullying intervention needs to be a cohesive effort that includes everyone [9, 57].

Interestingly, management participation in an intervention can enhance overall staff engagement, but also help with the way student bullying might be ‘understood’ and dealt with - some authors suggest bullying incidents might be better viewed as medical error, thereby avoiding any implication of a personal failure on the part of the bully [45], and allowing staff to engage better in an intervention focusing on good practice [41].

While a percentage figure is not offered, the literature also suggests including as many staff as possible within a department or section in an intervention, to enhance the effect on overall workplace culture [72]. As with the support offered by active learning processes in upskilling staff or changing behaviour, ‘saturation’ of new knowledge or skill has been said to increase staff confidence to implement what is learned [48, 54].

### Deleterious effects of intervention targeting

Under this theme we report further specific evidence to suggest that staff might suffer negative consequences of a bullying intervention. These findings add to the work of McGregor [34] about deleterious effects of policy, reported above. Studies explain how an intervention targeting a single professional group (e.g. ‘the nurses’) can hurt that group, by making the group feel ‘picked on’ and at fault [34]. Similarly, interventions specifically targeting the ‘bully,’ e.g. where mentioned in an intervention description (‘Let’s stop the bullies!’) also marginalise and infer guilt, even in the innocent. Such inferences have also been shown to result in a staff member’s failure to engage because they might respond with a self-protective ‘counter challenge.’ Some authors warn such responses to targeting can also lead to continued or renewed bullying behaviour [9, 34, 71, 73]. Such approaches underpin the incorrect assumptions that bullying lies within individuals unrelated to context and that punitive measures are better than supportive ones.

We also find reference to deleterious effects where the ‘staff bystander’ to student bullying is targeted. While one paper indicates bystander behaviour is an imperative part of bullying management [19] another raises a rather accusatory indication that the bystander who fails to act is somehow at fault for continued bullying: ‘doing nothing makes you part of the problem’ ([64], p. 299). Some authors also suggest staff should prevent bullying by proactively treating each other as potential bullies [66] – which we find a rather unproductive or harmful way to approach the treatment of one’s colleagues.

### Frame the intervention to improve behaviour, not eradicate bad behaviour

Research from the last decade steadily adds weight to earlier indications that both an intervention’s content and its mode of administration are important. A generally ‘positive’ focus aimed to improve behaviours can better effect behaviour change than one which is negative or punitive [15, 47, 50, 52, 71]. Thomas [16] and Thomson [71] both suggest such a positive focus is engaging and empowering for participants, and important to the eventual creation of a blame-free environment, again, similar to that described in the field of clinical error prevention. Similarly, Siassakos [73] and Schoonbeek & Henderson [51] report a positive focus on enhancing skills of teaching and learning can help bullying by positively influencing overall work culture.

### Interventionist teaching and facilitation skills matter

Having described facets of content, topic and intervention framing, we now describe evidence strongly suggesting that the skills of the interventionist can significantly influence its outcomes. This is echoed by what we find in the field of adult learning more generally. As with active learning processes, authors suggest administering a program or determining its content is no guarantee that it will be effective but will also depend on other factors. Jacobs and Bergen [42] offer a specific example from practice, about how an interventionist’s process and content might be appropriate, but their lack of skill can mean participants side-track discussion away from a central remit. In this case, the aim was primarily to aid personal reflection on workplace behaviour. Other authors [68, 74, 75] report that as part of skilful teaching, it is those skills relating to interventionist’s skills of confidentiality (e.g. how participants hear the teacher talk about others) that can specifically enhance staff engagement: several studies specifically cite confidentiality as key to staff participant engagement, especially where intervention ‘topics’ are highly personal. Examples given in the literature of ‘perceived confidentiality’ are the interventionist who offers confidentiality because they are independent of the participants’ employer [68, 69] or because they have simply formed a trusting, functional relationship with staff with whom they work (see also Theme 2).

## Discussion

### The information available in the literature presents challenges in review

The literature about ameliorating student bullying in the clinical workplace presents several challenges in review. Literature was both complex and patchy, making it difficult to develop a clear understanding of best practice. Details missing from the literature, as noted by others [27, 32], are often substantive, such as a lack of detail about an intervention or what is meant by a ‘workshop’. Ultimately, such omissions make interpretation and replication difficult [43, 44] and thus limit the total value of such work.

Many descriptions of interventions also lack detail as to attendee involvement and teaching methods, research methods, and results of any evaluation. Likewise, there can be lack of detail regarding the intervention group. Some interventions are administered to diverse populations and, while such diversity is important, the impact and effect may then vary depending on the staff and student roles in each.

We also note a lack of detail about how some outcomes are ascribed to an intervention [55], e.g. Jacobs & Bergen [72] report improved workplace ‘atmosphere’ after workshops, but without attendant explanation as to how atmosphere was reported or measured.

### Effectively engaging adult learners

The overall focus of much of the literature seems to be on how best to engage adult, qualified staff in learning new behaviours or skills. We note a focus on the efficacy of active learning methods and as part of this, creating a productive relationship with staff as ways to help them learn and improve the overall environment for students. All these factors are discussed in the more general literature about adult learning. Thus, we are offered ideas about a possible framework for planning a student bullying intervention that contrasts to a reactively imposed approach such as a lecture about behaviour, which might save time and money, but which could be seen to perpetuate negative work atmosphere or culture. We also note the emergence of the idea that active learning processes can offer staff the support they might need (say, if values change might be required) and the role of ongoing support from every member of the workforce subsequent to such learning.

### The review reveals *some* detail about affecting positive behaviour change

Specific themes identified here illustrate the importance of understanding the workplace experiences of staff, the nature of their work, incumbent stressors and how an effective intervention might then be framed and administered. We note the importance of including these factors in an intervention framed in ways to avoid ‘targeting’ a potential bully, or ‘blaming’ a specific staff group. The importance of policy for upholding behavioural standards is also noted, alongside a considerable consensus acknowledging the limits of policy in terms of actual positive behaviour change. As well as suggesting more effective ways to pitch an active learning intervention, we gain an understanding of how it should be staffed, to include indications of potential deleterious outcomes should the ‘wrong’ person administer it. This last finding is echoed in the higher education literature, which has now established a focus away from teaching ‘content’ to an understanding of better ‘process,’ to ‘who’ a teacher is [76].

### Catalysts, support and policy - issues dealt with incompletely

While identifying bullying catalysts has been highlighted as important for effective behaviour change, there is less guidance on what to do once they are identified. Further guidance about better managing these issues in the workplace management (perhaps as part of an intervention) would benefit from the addition of a synthesis of relevant literature about general clinical workplace management (e.g. workflow control for environmental issues) or professionalism and values development (for personal catalysts to bullying). Such a synthesis is outside the remit of our current work but seems important, given other evidence suggesting apparent lack of support from management in workplace issues can mean staff subsequently interpret, and reject, any intervention as a penalty, e.g. for ‘not coping’ [55]. There is also a growing understanding that neglecting to address bullying in the workplace might render management personally culpable [40, 41, 77].

The literature also contains little guidance about policy to specifically cater for student needs, for example how to help a student to report bullying ‘safely.’ Such a provision would likely improve reporting rates, as well as action taken on it. Instead, the present day complainant can still fear exacerbated retribution, to include serious academic consequences. We watch with interest about how programmes such as the Vanderbilt [78–80] cater to this unique and vulnerable population.

### Concern about bystander research

We are concerned about how the literature portrays the role of the staff bystander who says nothing as being complicit in bullying, potentially ‘targeting’ these (likely) innocent staff members. If the environment remains unsafe and broader issues are not addressed, a staff bystander making a ‘stand’ for the bullied student can be placed in considerable danger of being bullied themselves. Such dangers are described in recent media reports which suggest a bystander who reports bullying to management risks having their academic or career progression curtailed by similar means to that suffered by the student they were trying to help [26]. As we found with policy and reporting, there are few references to how a bystander may exactly be supported, or specify an appropriate process by which student bullying might be reported, here, by a person who is not the actual ‘target.’ We thus find some of the advice offered rather out of place, e.g. for a bystander to confront a bully [59], given that the main remit of any bullying intervention should be to encourage harmony, not discord, in a workplace. Instead, an approach focused more on the learning environment, not individuals, seems more likely to be effective.

### Limitations of our review methods

Results reported here are limited by the purposeful exclusion of articles about interventions exclusively about qualified staff (inter-staff bullying) which may have excluded useful insights into bullying interventions more generally. There is also a substantial literature from Norway about staff-staff interventions which might only be partially reflected in our use of the research-based book by Einarsen [9]. We also find a relative lack of specific foci on helping allied health professional students despite the inclusion of appropriate terms in our search criteria. Findings are also limited by lack of evaluation of results of anti-bullying interventions administered by private enterprises in the clinical environment, e.g. Vanderbilt programs (see Swiggart [22, 80], Yamada [81], Hickson, et al., [82], Webb et al. [78], Dubree et al. [78] for partial descriptions). Such enterprises offer interventions which aim to ameliorate inter-staff bullying but we find little specific reference to student bullying.

Despite these potential limitations, our review methods seem rigorous, were made explicit, and represent a novel approach compared with many other reviews, such as those which follow a ‘levels of evidence’ approach (e.g. Green [35]). Such difficulties are relevant because they are highly likely to be experienced by others charged with developing a bullying intervention. Instead, we offer concise, practical guidance with the aim to help ameliorate bullying for students worldwide.

### Summary of recommendations for further research

Despite the concerns and limitations described, we feel confident the themes identified offer robust areas for future interventions and research. As such, we identify the following as foci for further research, review or synthesis, in the context of student bullying. We suggest:

1. a specific focus on, and evaluation of, what works for students, in particular, regarding bullying in the clinical setting;
2. a specific focus on student-student bullying and any interplay between this literature/interventions with this in mind, and the current review;
3. a synthesis of ideas about the management of environmental and personal catalysts with those about identification and intervention;
4. the development and evaluation of a ‘safe’ system for a student, staff or bystander to raise a bullying issue or make a complaint;
5. a more in-depth understanding of relative effectiveness of active learning methods in interventions, how these might best be realised for values change and staff support, and any specific deleterious effects of learning methods which are less effective, e.g. lectures;
6. how the recidivist bully might be best engaged in an intervention to include research into how ‘the accused’ experiences bullying intervention, to add further to knowledge about ‘what not to do.’
7. the development and evaluation of methods to address recidivist bullies;
8. a more detailed understanding of the effect of ‘who an interventionist is’ on staff engagement and learning;
9. the deliberate avoidance of processes likely to be deleterious to staff undertaking a bullying intervention.

## Conclusion

We synthesise findings about interventions designed to reduce bullying of students in the clinical environment, into themes to guide the practitioner. We offer substantial synthesised important and useful information about what exactly might be done to help students in clinical practice. In doing so, we also bring together some emergent ideas about what we should ‘stop doing,’ some reassurance about aspects which seem to be developing in alignment with the literature about adult education and ways to avoid pitfalls and potential deleterious effects of an intervention. Ultimately, we aim this review to improve the learning and lives of our students, help staff in clinical practice better enjoy their work and maintain an increased overall quality of clinical service for patients.

## Additional file

Additional file 1: Appendix A Evidence table of literature used in the review and the corresponding theme(s) they contributed towards.* (DOCX 37 kb)

## Abbreviations

EMBASE: Excerpta Medica dataBASE, a biological and pharmacological database of international published literature
ERIC: Educational Resources Information Center, a database of international literature with a focus on education
NHS: National Health Service, a public healthcare provider based in the United Kingdom
PICO: Population, Intervention, Comparison, Outcome
