# Supplementary material for: Interventions addressing student bullying in the clinical workplace: a narrative review
Source: BMC Med Educ. 2019 Jun 21;19:220. doi: 10.1186/s12909-019-1578-y (PMC6588850; doi:10.1186/s12909-019-1578-y)
Supplement: Supplementary file 1 — Appendix A Evidence table of literature used in the review and the corresponding theme(s) they contributed towards.* (DOCX 37 kb) [file 12909_2019_1578_MOESM1_ESM.docx]

**Additional file 1: Appendix A - Evidence Table**

| **Reference** |  | **Article type** | **Sample - Participants** | **Design - Qualitative/Quantitative** | **Type of intervention used (if any)** | **Results/Conclusions/Key Points** | **Contribution to particular theme(s) in the results:** |
| --- | --- | --- | --- | --- | --- | --- | --- |
| Barnsteiner et al. (2001) | 56  3, 4 | Descriptive evidenced based article on the creation of a disruptive conduct policy | N/A | N/A | N/A | 1. Policies designed to create a healthy work environment include: assessing health of the environment, outlining standards & expectations, implementing systems & structures to support healthy environment including education of staff. 2. Important processes: raising awareness, legitimization via a particular committee structure, involving people expected to change if possible, specific dissemination and implementation involving education and training that involved all staff. | 3. Policy: necessary, but not sufficient  4. No targeting specific groups |
| Best et al. (2010) | 66  3 | Descriptive evidenced based article about working with sexual harassment and gender discrimination complaints | N/A | Describing a programme to handle complaints of possible episodes of sexual harassment or gender discrimination | N/A | 1. Facilitate separate in-person meetings between the complainant and the accused. 2. Flexibility in determining complaint outcomes. 3. Emphasise the importance of confidentiality and non-retaliation to all parties. 4. Strong wider institutional support for the Office of Gender Equity (OGE) that handles these complaints. 5. OGE staffed by clinical professionals with expertise in sexual harassment, sexual assault, and interpersonal violence. 6 Developed a reasonable but fixed time frame for resolving complaints. | 3. Policy: necessary, but not sufficient 5. Frame the intervention to improve behaviour. 6. Interventionist skill matters |
| Clark et al. (2013) | 50  5 | Research about using PBL scenarios to prepare nursing students to address incivility | Senior nursing students | Qualitative evaluative questionnaire | Evaluating students learning from participating in a problem-based scenario on incivility. | 1. 64% of responses mentioned they learned how to recognise and address incivility in nursing practice. 2. 13% of responses mentioned they gained self-awareness to cognizant of own behaviour and how to treat others. 3. 10% of responses mentioned they learned to be supportive, positive, and respectful. | 5. Frame the intervention to improve behaviour |
| Clarke et al. (2012) | 63 | Research investigating the state of bullying in clinical nursing education. | Undergraduate nursing students | Survey - Likert-scale questionnaire | N/A | 1. Institutions involved in the education and training of healthcare professionals have the responsibility for defining bullying and implementing policies and procedures that address the issue. 2. Extend health-care organisation non-violence policies to include nursing students. 3. Promulgate these policies as well as reporting mechanisms. | 3. Policy: necessary, but not sufficient |
| Cohen et al. (2005) | 72  6 | Descriptive evidenced based article outlining a support programme designed to help students and doctors with their communication skills. | Medical students and qualified doctors who were perceived to have communication difficulties. | N/A | Specific support tailored to individuals including activities geared to improved functioning at work. E.g. coaching with interprofessional communication, personality and communication style. | 1. This article describes a programme, no outcomes are reported. However, what was learned in developing the service was: a need to clarify boundaries and responsibilities of the programme, comfort for clients regarding confidentiality, importance of specific skills needed by the people running the programme. | 6. Interventionist skill matters |
| Felblinger (2009) | 39  1,3 | Book Chapter (synthesising published research) | N/A | N/A | N/A | 1. Particular organisational factors are known to facilitate bullying and disruptive behaviours in healthcare organisations. Including changing hierarchies, high levels of stress combined with the science changing daily and conflicting loyalties. | 1. Understand bullying catalysts  3. Policy: necessary but not sufficient |
| Fried et al. (2012) | 53  3 | Research on eradicating medical student mistreatment | Third-year medical students | Longitudinal study using a mixed methods survey across 4 time periods. | Created policies to prevent mistreatment. Instituted safe reporting of mistreatment, provided resources for discussion and resolution, and a comprehensive education programme targeting students, residents, and faculty. | 1. The anti-mistreatment education programme or the mandatory sexual harassment prevention training were not accompanied by a decrease in *overall* incidence of mistreatment. Yet, in *specific* areas, before 1998 55% of students reported being verbally mistreated and 43% reported power mistreatment. From 1999-2008 a decrease was seen in both reported forms of mistreatment, an average of 38% of students reported they were verbally mistreated and 31% reported power mistreatment. The incidence of reported sexual harassment remained stable with 13% of students reporting being a victim of sexual harassment. After 1998, incidence of ethnic mistreatment dropped significantly from 17% to 12%. | 3. Policy: necessary, but not sufficient |
| Hakojärvi et al. (2014) | 65  3 | Research on health care students’ personal experiences and coping with bullying in clinical training. | Finnish healthcare students | Qualitative - semi-structured on-line survey. Investigating how healthcare student's cope with bullying. | N/A | 1. Less than half of students bullied reported they shared their experience with a teacher. A major theme was called 'Coping with Bullying by Sharing Experiences'. 2. From student reports, some students didn't share experiences of bullying with their teacher/clinical instructor because they perceived that doing this would be useless. 3. Students who shared experiences of bullying with a teacher/clinical instructor, usually received emotional support, information, and help in the form of a bullying intervention (i.e. teachers intervening in the bullying situation). | 3. Policy: necessary, but not sufficient |
| Hamblin et al. (2015) | 21  1,3 | Research identifying common catalysts of incivility in hospitals | Hospital staff | Qualitative retrospective study using the hospital central reporting system | N/A (as identifying catalysts not implementing and intervention) | 1. Catalysts identified were 'Work Behaviour' (e.g. conflicts arising from unprofessional behaviour) and 'Work Organisation' (conflicts arising from limited resources and high workload). | 1. Understand bullying catalysts  3. Policy: necessary, but not sufficient |
| Heru (2003) | 47  2 | Descriptive evidenced based article outlining a programme that uses role playing to increase residents’ awareness of medical student mistreatment | Faculty involved in making the videotaped scenarios | N/A | Using videotaped mistreatment scenarios for role play sessions. | 1. Faculty involved in making the video tapes noted an increased ability to recognise mistreatment; previous unacknowledged personal mistreatment identified, improved ability to handle perceived mistreatment, exploration of the complexities of prejudice and the nature of difference, increased self-awareness and sensitivity to others, interest in the topic of boundaries and the teacher-learner relationship. | 2. Staff: understand what they do. |
| Heru (2014) | 48 | Discussion paper | N/A | N/A | Role-play | 1. Experiential learning is useful to understand how emotions affect behaviour or cognition. 2. Experiential teaching such as role play is an effective teaching method illustrating the effects of mistreatment. | 2. Staff: understand what they do. |
| Hills (2012) | 64  3,4 | Descriptive evidenced based article about what the medical practice employee needs to know about workplace bullying. | N/A | N/A | N/A | 1. Policies about behaviour are regularly ignored. A large percentage of bullying do not inform their employer (estimated at 40%). | 3. Policy: necessary, but not sufficient  4. No targeting specific groups. |
| Hollins-Martin and Martin (2010) | 57  3 | Descriptive evidenced based article about solutions to managers to decrease levels of bullying and harassment within maternity units. | N/A | N/A | N/A | 1. Managers have a responsibility to reduce bullying by constructing thoughtful policies and programmes. 2. Measuring to see if policies and programmes have been successful are important the BAHS measurement has been devised to do this. | 3. Policy: necessary, but not sufficient |
| Jacobs and Bergen (1995) | 40  2, 6 | Research report | Medical students and faculty | Pre- and post- surveys combined with an intervention | Educational retreats and workshops for faculty, workshops for students | 1.Results showed an improvement in school climate and decreases in gender-related problems  2. Need to be skilled in facilitation | 2. Staff: understand what they do  6. Interventionist skill matters |
| Jacobs and Bergen (2000) | 43  2  4 | Research about the impact of a programme to diminish gender insensitivity and sexual harassment at a medical school | Faculty and student development | Pre- and post- surveys combined with an intervention | Educational retreats and workshops for faculty, workshops for students | 1. Results indicated a significant improvement in the school environment following the intervention, as well as improvements in positive climate and cohesion scales and significant decrease in the degree to which faculty perceived sexual harassment, gender insensitivity, or gender discrimination to be problems. | 2. Staff: understand what they do.  4. No targeting specific groups. |
| Johnson (2011) | 58 | Descriptive evidenced based article - guide for intervention and research using an ecological model. | N/A | N/A | N/A | 1. Legislation does not make bullying disappear. Workplace bullying is a complicated problem. Using an ecological model helps to illustrate this complexity and provide a theoretical model when designing and testing an intervention. | 3. Policy: necessary, but not sufficient. |
| Johnston (1992) | 49  2 | Descriptive evidenced based paper on a programme to address insensitive behaviours toward medical students. | Faculty and students | N/A | Workshop containing video of vignettes of examples of insensitive behaviour accompanied with role-play and small group discussions. | 1. Reported use of putting what was learned into practice is evident (anecdotally) e.g. more willing to confront peers and colleagues regarding racial and sexual insensitivity. | 2. Staff: understand what they do. |
| Lachman (2009a) | 55  3 | Descriptive evidenced based article on the practical use of the nursing code of ethics (part 1) | N/A | N/A | N/A | 1. Policies, such as ethical codes of conduct, are essential in communicating and guiding professional expectations. Yet policy alone has little positive effect on behaviour. | 3. Policy: necessary, but not sufficient. |
| Lachman (2009b) | 60  3 | Descriptive evidenced based article on the practical use of the nursing code of ethics (part 2) | N/A | N/A | N/A | 1. Policies, such as ethical codes of conduct, are essential in communicating and guiding professional expectations. Yet policy alone has little positive effect on behaviour. | 3. Policy: necessary, but not sufficient. |
| Lasater et al. (2015) | 46  2  4  5 | Research about reducing incivility in the workplace | Variety of nursing staff spanning all levels | Three-phase education intervention; mixed methods approach. | 1. 1-hour didactic presentation 2. Four-hour role-play session 3. Two-hour simulation session | 1. Educational interventions can be effective in decreasing incidences of perceived incivility and increasing self-efficacy. 2. Post-intervention refreshers or intermittent unit discussions may increase the sustainability of collective self-effacement. | 2. Staff: understand what they do  4. No targeting specific groups  5. Frame the intervention to improve behaviour. |
| Leiper (2005) | 75  3 | Viewpoint | N/A | N/A | N/A | 1. What is involved in nursing 'horizontal violence': disenfranchising work practices, low self-esteem, general and hierarchical abuse. 2. The institution must have policies that are proactive, not reactive to identify and control circumstances where horizontal violence may occur. It must also have an action plan that is actionable that gives mangers the ability to step-in and act. | 1. Understand bullying catalysts  3. Policy: necessary, but not sufficient. |
| Longo (2013) | 19  1  2 | Descriptive evidenced based article about bullying and the older nurse. | N/A | N/A | N/A | 1. Hierarchical and disempowering work environments may be contributing to bullying. Initiatives involving empowering staff need to be developed for prevention. 2. Particular initiatives that recognise the skills and knowledge that contribute to patient care need to be developed. | 1. Understand bullying catalysts  2. Staff: understand what they do. |
| Lucey and Souba (2010) | 52  2  4  5 | Descriptive evidenced based article about the problem with professionalism. | N/A | N/A | N/A | 1. Re-thinking addressing failures in professionalism as a type of medical error may provide new ways of approaching professionalism amongst physicians. 2. This includes emphasising the fallibility of humans; the individual and collective responsibility of all physicians to accept the work required to sustain the values we espouse; and the importance of supportive cultures monitored for their impact on professionalism; as well continuous learning and improvement by all. | 2. Staff: understand what they do  4. No targeting specific groups  5. Frame the intervention to improve behaviour. |
| Martin (2008) | 1  2, 3 | Descriptive evidenced based article about the development of an evidenced-based framework to help hospital administrators to promote a safe working environment. | N/A | N/A | N/A | 1. Develop and adopt policies for disruptive behaviour and enforce the policy consistently throughout the organisation. 2. Create and sustain a high-performance work culture that focuses on attaining organisational goals by enabling individuals & groups at all levels to maximise potential. 3. Identify and reward behaviours that demonstrate collaboration, respect, and interpersonal ethics. 4. For potential victims of disruptive behaviour, employers should train workers about their rights and responsibilities. 5. For the disruptive individual, experiential learning activities such as simulations, role-playing, and case studies to be used to maximise transfer of learning to the work environment. | 2. Understand what staff do  3. Policy: necessary, but not sufficient 5. Frame the intervention to improve behaviour. |
| McGregor (2015) | 32  1, 2, 3,4 | Book chapter (synthesising published research) | N/A | N/A | N/A | 1. Institutions aware of bullying antecedents can breed bullies, and therefore institutional managers need to be able to put in place extra support and resources in dealing with these antecedents. 2. The organisation needs to be mindful of the ongoing relationship with victims of bullying as well as alleged bullies. 3. Ongoing support for the targets of bullying and the alleged bullies is necessary to maintain this relationship. 4. Policies addressing bullying and harassment should be utilized. 5. Identifying and targeting alleged bullies can result in failure to engage them in any way especially if the idea is to help them improve their behaviour. | 1. Understand bullying catalysts  2. Staff: understand what they do  3. Policy: necessary, but not sufficient  4. No targeting specific groups |
| Moscarello et al. (1996) | 73  2 | Research on the impact of faculty education on the incidence of sexual harassment experienced by medical students | Fourth year medical students | Pre-and post-surveys with an intervention | Seminars, workshops and lectures on sexual harassment for faculty members and students | 1. The authors found a decrease in sexual harassment from 35.2% in 1991 to 22% in 1994. There was a decline in in non-contact sexual harassment, however contact harassment remained mostly unchanged. | 2. Staff: understand what they do |
| Robinson and Stewart (1996a) | 41  2 | Descriptive evidenced based article about a curriculum on physician-patient sexual misconduct and teacher-learner mistreatment (part 1) | Staff and students | N/A | Faculty development lectures | N/A (see part 2 below) | 2. Staff: understand what they do |
| Robinson and Stewart (1996b) | 42  2 | Descriptive and evaluative evidenced based article about a curriculum on physician-patient sexual misconduct and teacher-learner mistreatment (part 2) | Staff and students of various healthcare professions | N/A | Workshop - discussing case vignettes | 1. Of participants who completed an evaluation of the course (lectures plus discussion of case vignettes): 39% said they were likely to change their clinical or teaching practices and 38% said they already practiced in a manner congruent with what was modeled. | 2. Staff: understand what they do |
| Rosenstein (2009) | 37  1  2 | Viewpoint discussing early intervention helping to prevent disruptive behaviour | N/A | N/A | N/A | 1. Having the right policies and procedures in place is part of the 'right thing to do'. 2. Disruptive events are more likely to happen in high stress/intensity areas. 3. Get alongside the physician to understand the physician's world in order to provide coaching and counselling. | 1. Understand bullying catalysts  2. Staff: understand what they do |
| Rosenstein (2015) | 38  1, 2,3,4 | Descriptive evidenced based article about addressing the causes and consequences of disruptive behaviours in healthcare setting | N/A | N/A | N/A | 1. Many external and internal factors contribute to disruptive behaviours. 2. A strong contributor is stress and burnout in healthcare professions. 3. Solutions involved raising awareness, addressing barriers, involving all staff in addressing the issue, suitable policies to promote appropriate professional behaviours. | 1. Understand bullying catalysts  2. Staff: understand what they do  3. Policy: necessary, but not sufficient  4. No targeting specific groups. |
| Schoonbeek and Henderson (2011) | 51  2,4, 5 | Descriptive evidenced based article about shifting workplace behaviour to inspire learning | Nursing staff | N/A | Staff development with a variety of techniques used: role-play and role modelling. Reward system for staff also used. | 1. Creation of a learning environment through staff development to combat bullying. 2. Building learning cultures fundamentally requires a safe psychological environment where bullying is not tolerated. 3. The creation of training environments cannot be imposed - but be developed with team members. 4. A variety of strategies to promote learning in practice along with reward and recognition is needed to promote learning activities. | 2. Staff: understand what they do  4. No targeting specific groups  5. Frame the intervention to improve behaviour. |
| Siassakos et al. (2009) | 70  4,5 | Research about an evaluation of a strategy to improve undergraduate experience in obstetrics and gynaecology | Medical students - Year 4 | pre-testing - intervention - post-testing with questionnaire | Interprofessional experiences. Focus on working relationships as opposed to ad hoc learning exercises. Simulation-based obstetric emergency training also given. Hands-on experience is becoming more difficult to access. | 1. Interprofessional relationships improved after the O&G attachment. 2. Some improvement in professional teamwork and communication. 3. Authors did not witness any bullying or interprofessional difficulties. | 4. No targeting specific groups.  5. Frame the intervention to improve behaviours |
| Souba et al. (2011) | 66  3 | Research about problems that impair performance but which the community does not discuss called ‘elephants’ | Chairs of departments of medicine and of surgery at an Academic Health Centre (AHC). | Survey assessing the prevalence of organisational silence | N/A | 1. AHC 'elephants' (obvious problems that impair performance) are prevalent and detrimental to learning, decision making, and morale. 2. This culture of silence has a tendency to covertly ignore many of the upsetting issues. | 3. Policy: necessary, but not sufficient. |
| Thomas (2010) | 15  1,3 | Descriptive evidenced based article about teaching student & registered nurses strategies to deal with violent behaviours in the professional practice environment | N/A | N/A | N/A | 1. Faculty to communicate to nursing students they are important.2. Sign a violence-free behaviour contract (between students and staff) to help establish accountability. 3. Educate faculty and students during an orientation to outline appropriate interventions such as cognitive recognition, reflective journaling, care fronting, recognizing personal anger triggers. | 1. Understand bullying catalysts  3. Policy: necessary but not sufficient |
| Thomson et al. (2015) | 71  5,6 | Research exploring the experiences and implementing strategies for physiotherapy students who perceive bullying or harassment on clinical placements. | Five final year physiotherapy students who self-declared instances of bullying and harassment | Participatory action research (via focus groups) - exploring strategies to help the bullying and harassment of physiotherapy students. | Focus groups | 1. Analysis suggested certain tools of coping strategies such as: trying to understand the educator’s 'foibles', use assertiveness, harnessing wider support such as a visiting tutor. 2. Lastly, wider support from the University senior staff should be clearly documented. | 5. Frame the intervention to improve behaviour. 6. 6.  6.Interventionist skill matters. |
| Trépanier et al. (2016) | 36  6 | Review of work environment antecedents of bullying regarding registered nurses | Nurses | Narrative review | N/A | 1. Workplace bullying was positively correlated to workload, organisational constraints, bad and stressful work situations and negatively correlated to job control, promotional opportunities, reward and structural empowerment. | 1. Understand bullying catalysts. |
| Walton (2015) | 61  3 | Descriptive evidenced based article about sexual equality, discrimination and harassment in medicine | N/A | N/A | N/A | 1. Policy alone will not change culture, must be accompanied by strong action. 2. Token attention to complaints made without removing teachers who behave unprofessionally reinforces belief that nothing will change. 3. Acknowledging power influence of supervisors on learning outcomes is crucial. | 3. Policy: necessary, but not sufficient. |
| Zweibel and Goldstein (2001) | 43  2 | Research about transferring conflict resolution training from the workshop to the workplace | Faculty physicians and scientists | Qualitative - including pre-post workshop surveys; anonymous feedback; observer field notes; post workshop focus group; semi-structured interviews | 2-day workshop | 1. After 1 year of participating in the workshop, participants report applying conflict-resolution skills to difficult situations. 2. Participants gained the perspective that conflict doesn't need to be overwhelming and destructive. 3. Participants gained confidence to approach conflict systematically, rather than just reacting to events. | 2. Staff: understand what they do. |

**APPENDIX A**

**Table 2: Evidence table of literature used in the review and the corresponding theme(s) they contributed towards**.*

*N/A = Not Applicable
